# Supplementary material for: Characterizing quantum circuits with qubit functional configurations
Source: Sci Rep. 2023 Apr 4;13:5539. doi: 10.1038/s41598-023-31980-3 (PMC10073272; doi:10.1038/s41598-023-31980-3)
Supplement: Supplementary file 1 — Supplementary Information. [file 41598_2023_31980_MOESM1_ESM.pdf]

Supplementary information: Characterizing quantum circuits with qubit functional configurations

Zixuan Hu and Sabre Kais*

Department of Chemistry, Department of Physics, and Purdue Quantum Science and Engineering Institute, Purdue University, West Lafayette, IN 47907, United States
**Email:* kais@purdue.edu

S1. The qubit functional configuration layers of the quantum Fourier transform circuit.

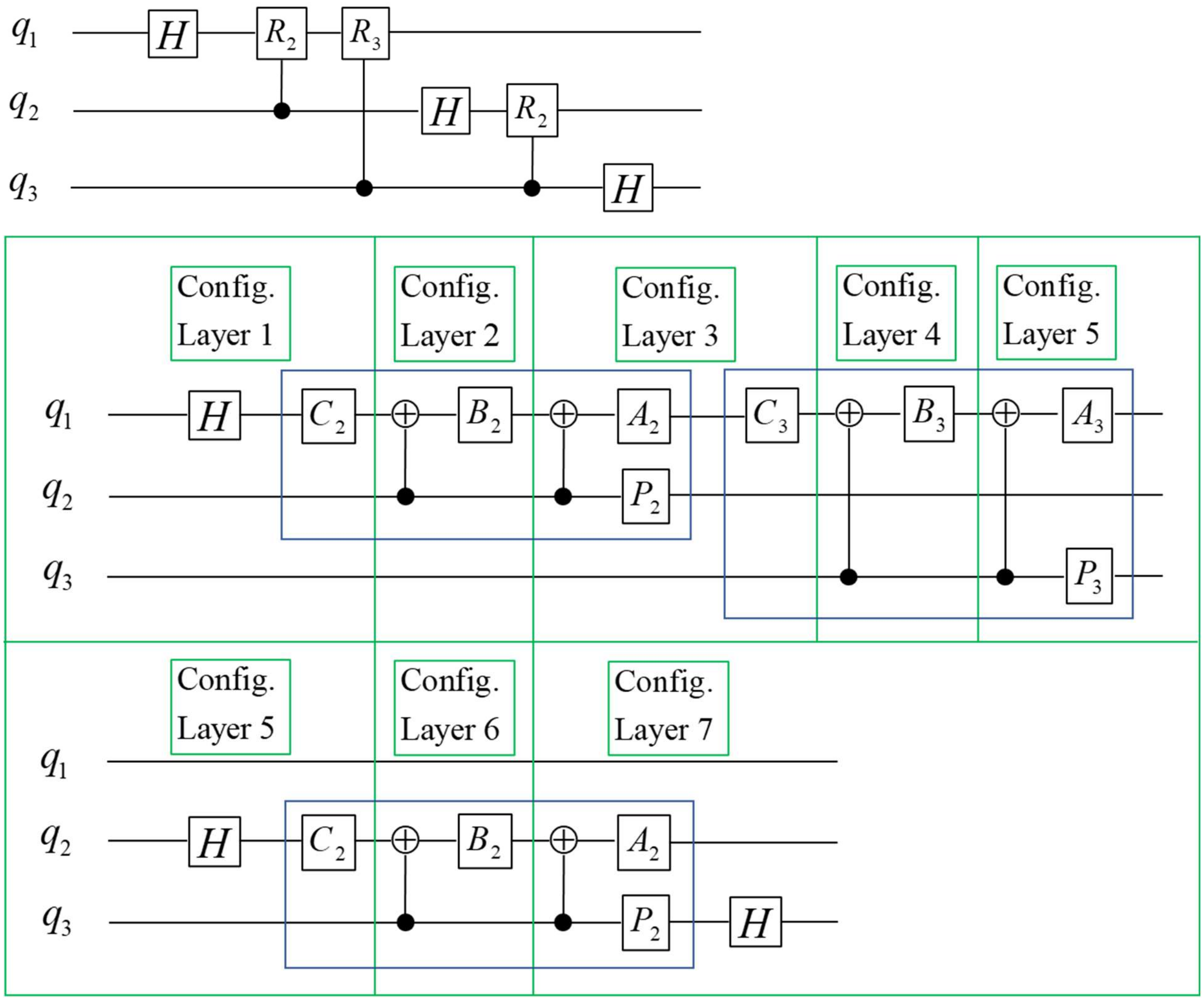

Figure S1. The 3-qubit quantum Fourier transform (QFT) circuit (top) analyzed with the functional configuration picture (bottom). The controlled-R_h gates are decomposed into CNOT gates and 1-qubit unitaries as enclosed in the blue boxes (see the description below for the details of these gates). The functional configuration layers are indicated by the green boxes. There are 7 layers in total, with each layer after the 1st one defined by a single CNOT gate.

In the main text we have applied the functional configuration theory to the hardware-efficient ansatzes of variational quantum algorithms. For the ansatzes all the layers have the same functional configuration and hence we have only studied one layer. In this section we study the quantum Fourier transform (QFT) circuit that has different functional configurations on different layers, such that the circuit is described by a sequence of layers. The QFT [1] is an essential subroutine in many important quantum algorithms such as the phase estimation algorithm [2] and Shor's factorization algorithm [3]. Here we analyze the functional configuration sequence of a 3-qubit QFT circuit:

In Figure S1 we first decompose the controlled-R_h gates, defined by $R_h = \begin{pmatrix} 1 & 0 \\ 0 & \exp(2\alpha_h i) \end{pmatrix}$ with $\alpha_h = \frac{2\pi}{2^{h+1}}$, into CNOT gates and 1-qubit unitaries as enclosed in the blue boxes. The standard decomposition [1] has $A_h = R_z\left(\frac{2\pi i}{2^h}\right)$, $B_h = C_h = R_z\left(\frac{-2\pi i}{2^{h+1}}\right)$, and $P_h = \begin{pmatrix} 1 & 0 \\ 0 & \exp(i\alpha_h) \end{pmatrix}$. The decomposed circuit on the bottom of Figure S1 contains 7 functional configuration layers as indicated by the green boxes, and the functional configuration sequence is: $(q_1,q_2,q_3) \to (q_1 \oplus q_2, q_2, q_3) \to (q_1 \oplus q_2, q_2, q_3) \to (q_1 \oplus q_3, q_2, q_3) \to (q_1 \oplus q_3, q_2, q_3)$ $\to (q_1, q_2 \oplus q_3, q_3) \to (q_1, q_2 \oplus q_3, q_3)$. In this particular circuit the 1st configuration layer is the initial configuration of (q_1,q_2,q_3), while each configuration layer after the 1st one is defined by a single CNOT gate, such that the number of CNOT gates is equal to the number of layers minus one. Considering the standard result of having $\frac{n^2-n}{2}$ controlled-R_h gates for an n-qubit system [1], and each controlled-R_h gate being decomposed into two CNOT gates, the total number of functional configuration layers is n^2-n+1 for an n-qubit QFT circuit. We see the number of layers scales as $O(n^2)$ and thus agrees with the well-known gate scaling of the QFT circuit [1] – as detailed in the main text Section 2.5, in general the functional configuration picture captures the important property of circuit complexity.

S2. Equivalence between qubit functional configurations due to permutation of functionals.

In the main text we mentioned that if there is only one layer of functional configuration, and two functional configurations contain two permutations of the same collection of functionals, then the two configurations can be considered as equivalent. Below we give a concrete example of a 3-qubit system. Consider the two functional configurations of $C_1 = (f_1 = q_1, f_2 = q_2, f_3 = q_3)$ and $C_2 = (f_1 = q_1, f_2 = q_3, f_3 = q_2)$. Clearly C_2 can be obtained from C_1 by swapping f_2 and f_3. By studying the effects of CNOT gates on modifying functional configurations, the swap between f_2 and f_3 can be achieved by applying sequentially $\text{CNOT}_{2\to3}$, $\text{CNOT}_{3\to2}$, and $\text{CNOT}_{2\to3}$ to

$C_1 = (f_1 = q_1, f_2 = q_2, f_3 = q_3)$. To see this is correct, the configuration after the 1st $\text{CNOT}_{2\to3}$ is $(f_1 = q_1, f_2 = q_2, f_3 = q_2 \oplus q_3)$, after $\text{CNOT}_{3\to2}$ is $(f_1 = q_1, f_2 = q_3, f_3 = q_2 \oplus q_3)$, and after the 2nd $\text{CNOT}_{2\to3}$ is $C_2 = (f_1 = q_1, f_2 = q_3, f_3 = q_2)$. Now suppose the initial state vector is $|\varphi_1\rangle = \sum_{i=0}^{7} a_i |i\rangle$, then we have:

$$\varphi_1 = \begin{matrix}000\\001\\010\\011\\100\\101\\110\\111\end{matrix}\begin{pmatrix}a_0\\a_1\\a_2\\a_3\\a_4\\a_5\\a_6\\a_7\end{pmatrix} \xrightarrow{\text{CNOT}_{2\to3}} \begin{matrix}000\\001\\010\\011\\100\\101\\110\\111\end{matrix}\begin{pmatrix}a_0\\a_1\\a_3\\a_2\\a_4\\a_5\\a_7\\a_6\end{pmatrix} \xrightarrow{\text{CNOT}_{3\to2}} \begin{matrix}000\\001\\010\\011\\100\\101\\110\\111\end{matrix}\begin{pmatrix}a_0\\a_2\\a_3\\a_1\\a_4\\a_6\\a_7\\a_5\end{pmatrix} \xrightarrow{\text{CNOT}_{2\to3}} \varphi'_1 = \begin{matrix}000\\001\\010\\011\\100\\101\\110\\111\end{matrix}\begin{pmatrix}a_0\\a_2\\a_1\\a_3\\a_4\\a_6\\a_5\\a_7\end{pmatrix} \quad \text{S(1)}$$

where the φ_1 vector is in the configuration of $C_1 = (f_1 = q_1, f_2 = q_2, f_3 = q_3)$ and the φ'_1 vector is in the configuration of $C_2 = (f_1 = q_1, f_2 = q_3, f_3 = q_2)$. Clearly, φ'_1 compared to φ_1 is just swapping the entry a_1 for $|001\rangle$ with a_2 for $|010\rangle$, and swapping a_5 for $|101\rangle$ with a_6 for $|110\rangle$ -- this is just swapping q_2 and q_3. This means that applying a 1-qubit unitary on q_2 (or q_3) for φ_1 is equivalent to applying the same unitary on q_3 (or q_2) for φ'_1, and therefore $C_1 = (f_1 = q_1, f_2 = q_2, f_3 = q_3)$ is equivalent to $C_2 = (f_1 = q_1, f_2 = q_3, f_3 = q_2)$ in the sense that they contain the quantum circuits that perform equivalent operations. Therefore swapping functionals in the configuration leads to equivalent configurations. Now by the theory of permutation groups, any arbitrary permutation can be realized by a series of swaps, then we conclude that any configuration generated by permuting the functionals in the original configuration is equivalent to the original as both configurations have equivalent rules for the 1-qubit unitaries up to a permutation. This equivalence leads to the $n!$ term on the denominator in Equation (6) of the main text.

S3. Multiple different CNOT gate sequences producing the same functional configuration and the minimal gate sequence.

In the main text we have discussed the situation where multiple different CNOT gate sequences can produce the same functional configuration and thus the same total unitary operation. Here we present a concrete example and discuss how a minimal gate sequence can be defined with the functional configuration picture. Again consider a 3-qubit system, the configuration of $(f_1 = q_1 \oplus q_2 \oplus q_3, f_2 = q_1 \oplus q_2, f_3 = q_1)$ can be created from the initial configuration of

$\left(f_1 = q_1, f_2 = q_2, f_3 = q_3\right)$ by the CNOT sequence of $\left(\mathrm{CNOT}_{1\to2},\ \mathrm{CNOT}_{2\to3},\ \mathrm{CNOT}_{1\to3},\ \mathrm{CNOT}_{3\to1},\ \mathrm{CNOT}_{1\to3}\right)$. However the same configuration can also be created from the initial configuration by another CNOT sequence of $\left(\mathrm{CNOT}_{2\to3},\ \mathrm{CNOT}_{3\to1},\ \mathrm{CNOT}_{1\to2},\ \mathrm{CNOT}_{3\to2},\ \mathrm{CNOT}_{1\to3}\right)$. We see that these two CNOT sequences are equivalent in the sense that they produce exactly the same functional configuration. In fact, there can be more equivalent CNOT sequences such as $(\mathrm{CNOT}_{3\to1},\ \mathrm{CNOT}_{1\to2},\ \mathrm{CNOT}_{2\to3},\ \mathrm{CNOT}_{2\to1},\ \mathrm{CNOT}_{3\to1}, \mathrm{CNOT}_{1\to2},\ \mathrm{CNOT}_{2\to1},\ \mathrm{CNOT}_{1\to2},$ $\mathrm{CNOT}_{2\to3},\ \mathrm{CNOT}_{3\to2},\ \mathrm{CNOT}_{2\to3})$, which is longer than the previous two but still produce the same functional configuration. A major benefit of the functional configuration picture is it allows us to see the equivalence of these different CNOT sequences with ease, because they all correspond to the same functional configuration. Unlike gate sequences, types defined by functional configuration sequences uniquely characterize the corresponding quantum circuits.

This fact also allows us to define and find a minimal gate sequence for any given quantum circuit, because among all the equivalent gate sequences for any functional configuration, there must be a sequence or multiple sequences with the minimal number of gate count, and this sequence(s) will be the minimal required to create a part of the quantum circuit involving such a functional configuration. In fact if a gate sequence decomposition (may not be the minimal) is known for an arbitrary quantum circuit, for example the one described above: $(\mathrm{CNOT}_{3\to1},\ \mathrm{CNOT}_{1\to2},\ \mathrm{CNOT}_{2\to3},\ \mathrm{CNOT}_{2\to1},\ \mathrm{CNOT}_{3\to1}, \mathrm{CNOT}_{1\to2},\ \mathrm{CNOT}_{2\to1},\ \mathrm{CNOT}_{1\to2},$ $\mathrm{CNOT}_{2\to3},\ \mathrm{CNOT}_{3\to2},\ \mathrm{CNOT}_{2\to3})$, we can obtain the functional configuration for each layer based on this sequence (only one layer for this example): $\left(f_1 = q_1 \oplus q_2 \oplus q_3, f_2 = q_1 \oplus q_2, f_3 = q_1\right)$, and then find the minimal sequence to generate the same functional configuration: $\left(\mathrm{CNOT}_{1\to2},\ \mathrm{CNOT}_{2\to3},\ \mathrm{CNOT}_{1\to3},\ \mathrm{CNOT}_{3\to1},\ \mathrm{CNOT}_{1\to3}\right)$, and this will be a much shorter circuit that performs the same task. If there are multiple layers, joining the minimal sequence for each layer together and inserting the appropriate 1-qubit unitaries will then yield the overall minimal sequence for the quantum circuit. Therefore by the functional configuration picture we have a systematic way of defining and finding the minimal sequence of any quantum circuit.

S4. Characterizing practical properties of quantum circuits

In Equations (4) and (5) in the main text we have focused on the structural differences between the two qubit functional configurations. Here we discuss how such differences would translate into circuit properties when used in conjunction with the "unitary dependence theory" proposed in our recent study [4].

The current theory is mainly based on the CNOT gates as they perform additions on qubits to form the qubit functional configurations. On the other hand, the unitary dependence theory is mainly based on the 1-qubit unitary gates as they create probabilistic dependences on the qubits. Briefly summarizing, the unitary dependence theory provides a complete picture of how quantum gates

manipulate qubits and determine their measurement probabilities. For example, two qubits sharing dependences on certain 1-qubit unitaries will be dependent in measurement probabilities; and two qubits not sharing any dependences on 1-qubit unitaries will be independent in measurement probabilities. From the perspective of circuit design, a 1-qubit unitary shared by multiple qubits will allow collective manipulation of all qubits involved; while a 1-qubit unitary belonging to only 1 qubit will allow qubit-specific manipulations.

As detailed in the study of the unitary dependence theory [4], to generate the unitary dependence picture we need to figure out how CNOT gates transfer unitary dependences around the qubits, and this can only be done by using the qubit functional configuration theory in the current work. Consider the same ansatzes in Figures 2 and 3 of the main text and their corresponding qubit functional configurations in Equations (4) and (5) of the main text, the configuration in Equation (4) shows a more interconnected qubit structure than that in Equation (5). Based on these two qubit functional configurations we can then generate the respective unitary dependence pictures [4]:

The unitary dependence picture of the ansatz in Figure 2 of the main text

$$\left(q_1:\{U_2\sim U_6\},\ q_2:\{U_1,U_2\},\ q_3:\{U_1\sim U_3\},\ q_4:\{U_1\sim U_4\},\ q_5:\{U_1\sim U_5\},\ q_6:\{U_1\sim U_6\}\right) \qquad \text{S(2)}$$
$$\left(U_1:\{q_2\sim q_6\},\ U_2:\{q_1\sim q_6\},\ U_3:\{q_1,q_3\sim q_6\},\ U_4:\{q_1,q_4\sim q_6\},\ U_5:\{q_1,q_5,q_6\},U_6:\{q_1,q_6\}\right)$$

The unitary dependence picture of the ansatz in Figure 3 of the main text

$$\left(q_1:\{U_1,U_2\},\ q_2:\{U_2\},\ q_3:\{U_1\sim U_3\},\ q_4:\{U_2,U_4\},\ q_5:\{U_4\sim U_6\},\ q_6:\{U_6\}\right) \qquad \text{S(3)}$$
$$\left(U_1:\{q_1,q_3\},\ U_2:\{q_1\sim q_4\},\ U_3:\{q_3\},\ U_4:\{q_4,q_5\},\ U_5:\{q_5\},U_6:\{q_5,q_6\}\right)$$

In Equations S(2) and S(3), the 1st line of each unitary dependence picture lists all the qubits q_1 through q_6 and what 1-qubit unitaries affect each qubit; the 2nd line of each picture lists all the 1-qubit unitaries U_1 through U_6 and what qubits are affected by each unitary. We see indeed the more interconnected qubit structure in Equation (4) of the main text leads to the unitary dependence picture in Equation S(2) where all qubits depend on multiple unitaries and all unitaries affect multiple qubits – this means the qubits of the ansatz in Figure 2 of the main text are more dependent when measured and can only be collectively manipulated by varying the parameters of the 1-qubit unitaries. On the other hand, the more isolated qubit structure in Equation (5) of the main text leads to the unitary dependence picture in Equation S(3) where q_2 and q_6 each depends on only one unitary, and U_3 and U_5 each belongs to only one qubit – this means the qubits of the ansatz in Figure 2 of the main text are more independent when measured and can be manipulated more individually by varying the parameters of the 1-qubit unitaries. These examples show the structural properties of the qubit functional configurations can lead to practical properties of the ansatzes such as the measurement probabilities of the qubits and how the qubits are collectively manipulated.

1. Nielsen, M.A. and I.L. Chuang, *Quantum Computation and Quantum Information: 10th Anniversary Edition*. 2011: Cambridge University Press. 708.

2. Kitaev, A.Y., *Quantum computations: algorithms and error correction.* Russ. Math. Surv., 1997. **52**: p. 1191.
3. Shor, P.W., *Polynomial-Time Algorithms for Prime Factorization and Discrete Logarithms on a Quantum Computer.* SIAM J. Comput., 1997. **26**(5): p. 1484–1509.
4. Hu, Z. and S. Kais, *The unitary dependence theory for characterizing quantum circuits and states.* Communications Physics, 2023. Accepted.
